# Use of ellipsoid zone width for predicting visual prognosis after cataract surgery in patients with retinitis pigmentosa

**DOI:** 10.1038/s41433-021-01878-3

**Published:** 2022-01-01

**Authors:** Daiki Sakai, Seiji Takagi, Yasuhiko Hirami, Makoto Nakamura, Yasuo Kurimoto

**Affiliations:** 1 Department of Ophthalmology, Kobe City Eye Hospital, Kobe, Japan; 2 Department of Ophthalmology, Kobe City Medical Center General Hospital, Kobe, Japan; 3 Department of Surgery, Division of Ophthalmology, Kobe University Graduate School of Medicine, Kobe, Japan; 4 Department of Ophthalmology, Toho University Graduate School of Medicine, Tokyo, Japan

**Keywords:** Lens diseases, Retinal diseases

## Abstract

**Objective:**

To predict the visual prognosis of cataract surgery in patients with retinitis pigmentosa by measuring ellipsoid zone (EZ) width using spectral-domain optical coherence tomography.

**Methods:**

This retrospective study included patients with retinitis pigmentosa who underwent uncomplicated cataract surgery between December 2017 and June 2020. Preoperative best-corrected visual acuity (BCVA) and the best postoperative BCVA during follow-up were collected. EZ width was measured on preoperative cross-sectional optical coherence tomography images along the horizontal/vertical meridian through the fovea.

**Results:**

Thirty-eight eyes of 38 patients (22 female; mean [±standard deviation] age, 62.1 ± 11.8 years) were included. The median preoperative logarithm of the minimum angle of resolution BCVA of 0.52 (range, 0.00–3.00) significantly improved to 0.07 (range, −0.18–3.00) after surgery (*P* < 0.001). On preoperative spectral-domain optical coherence tomography images, the median horizontal, vertical, and average EZ widths were 783 (range, 0–9837), 761 (range, 0–10 250), and 769 (range, 0–10 043) μm, respectively. Postoperative BCVA significantly correlated with the horizontal (*r* = −0.784, *P* < 0.001), vertical (r = −0.777, *P* < 0.001), and average EZ widths (*r* = −0.777, *P* < 0.001). The area under the receiver operating characteristic curve for the ability of the horizontal, vertical, and average EZ widths to discriminate eyes with and without postoperative BCVA ≤ 0.3 was 0.971, 0.960, and 0.963, respectively, with best cut-off values of 513, 608, and 515 μm, respectively.

**Conclusions:**

EZ width measurement can help predict the visual prognosis of cataract surgery in patients with retinitis pigmentosa. A preferable visual acuity prognosis can be expected in patients with an EZ width of approximately 600 μm.

## Introduction

Retinitis pigmentosa (RP) is the most common hereditary retinal dystrophy, with a worldwide prevalence of approximately 1 in 4000 individuals [1]. The clinical course of RP is characterised by night blindness and progressive loss of the visual field due to the degeneration of rod photoreceptors. Although central vision is initially well preserved, visual acuity (VA) is ultimately impaired due to the degeneration of cone photoreceptors in advanced stages.

Cataract is a representative treatable complication of RP, which causes central vision impairment. Although cataract surgery has been reported to be beneficial for patients with RP [2–5], some concerns such as the specific postoperative complications, including posterior capsule opacification and anterior capsule contraction [2, 6], or the risk of phototoxic retinal damage do exist. The most important concern, however, is the poor predictability of postoperative VA because of the coexistence of lenticular and retinal pathologies. In fact, previous studies have reported that postoperative VA did not improve in some populations of patients with RP who underwent cataract surgery [2–5].

The development of spectral-domain optical coherence tomography (SD-OCT) has enabled a more detailed evaluation of retinal microstructures and has become the gold standard for the assessment of various retinal diseases. The second hyperreflective band observed on SD-OCT is referred to as the ellipsoid zone (EZ). The EZ status is considered to reflect photoreceptor function and is used as an indicator of visual outcomes in many retinal diseases [7]. Nakamura et al. reported that the integrity of the EZ line was important for the prediction of visual prognosis after cataract surgery in patients with RP [4]. The presence of a normal EZ line is naturally associated with a preferable prognosis, and disruption of the EZ line is associated with a worse prognosis.

In patients with RP, EZ disruption begins in the peripheral retina and advances centripetally towards the fovea [8]. Recently, measurement of the length of the EZ line (EZ width) has attracted attention because of its ease and reliability [9]. Because it helps to recognise the edge of the EZ line as the boundary between the healthy and unhealthy retina, the EZ width has been proposed as a good biomarker of retinal degeneration in patients with RP [8, 10]. Careful monitoring of the concentric centripetal progression of RP may be possible by quantitatively measuring the shortening of EZ width.

Although retinal dystrophy is progressive and irreversible, the management of treatable complications is important to preserve the best visual function in patients with RP at each stage. We should keep in mind that timely intervention is crucial for treating cataract in patients with RP to avoid overlooking a valuable opportunity for vision improvement. Thus, an objective indicator of postoperative visual function is required to determine the optimal timing of the intervention. The purpose of this study was to predict the visual prognosis of cataract surgery in patients with RP using EZ width measurement and to investigate the cut-off value of EZ width to determine whether the patients were expected to achieve a preferable prognosis.

## Materials and methods

### Study design

This retrospective study was performed according to the tenets of the Declaration of Helsinki and was approved by the medical ethics committee of Kobe City Medical Centre General Hospital (Kobe, Japan). The committee waived the requirement for informed consent for this observational study, which involved the use of medical records. The confidentiality of patient data has been maintained.

### Patients

We enroled 103 eyes of 64 patients with RP who underwent uncomplicated cataract surgery between December 2017 and June 2020 at the Kobe City Eye Hospital. Patients were diagnosed with RP on the basis of the clinical history, appearance of the fundus, visual fields, and full-field electroretinogram results. We excluded 43 eyes because of short follow-up (within 1 month) (8 eyes), unavailable preoperative OCT image (within 6 months) (14 eyes), poor OCT image quality (7 eyes), and comorbid macular diseases, including epiretinal membrane, cystoid macular oedema, vitreomacular traction syndrome, and lamellar macular hole (14 eyes). In addition, if both eyes of one patient were eligible (22 patients), one eye from each patient was randomly selected to account for inter-eye correlation [11]. Finally, 38 eyes from 38 patients were included in the study.

### VA measurements

Preoperative best-corrected visual acuity (BCVA) and the best postoperative BCVA during follow-up were collected. BCVA was obtained using Landolt C charts and then converted to the logarithm of the minimum angle of resolution (logMAR) equivalent for statistical comparisons. VA of hand movement was assigned as the equivalent of 3.0 logMAR units [12].

### SD-OCT evaluation

SD-OCT images were acquired using Spectralis (Heidelberg Engineering, Heidelberg, Germany). EZ widths in each eye were measured using preoperative OCT images. Cross-sectional OCT images along the horizontal/vertical meridian through the fovea were evaluated for this purpose. EZ width was defined as the distance between the temporal and nasal borders on the horizontal image or the superior and inferior borders on the vertical image of the EZ, where the EZ line disappeared. If the entire length of the EZ line exceeded the size of the OCT image, the borders of the EZ were set to be those of the OCT image [13]. In addition to the horizontal and vertical EZ widths, which were acquired from the horizontal and vertical OCT images, respectively, the average EZ width was calculated by averaging the horizontal and vertical EZ widths. The measurement of EZ width was performed twice by one observer (DS), and the average EZ width was calculated at each time of measurement. The mean values of the three parameters (horizontal, vertical, and average EZ widths) at each measurement were used for analysis. In addition, the presence of EZ disruption and disorganisation of retinal inner layers (DRIL) [14] was recorded in each eye. EZ disruption was defined as the presence of disruption of the remnant EZ line on the horizontal or vertical OCT images. EZ disruption was also considered as positive if the EZ line was not visible. DRIL was defined as the inability to identify the boundaries between the ganglion cell-inner plexiform layer complex, inner nuclear layer, and outer plexiform layer within the 1000-μm diameter of fovea on the horizontal or vertical OCT images [14, 15]. The presence of EZ disruption and DRIL was determined by agreement between two observers (DS and YH). All measurements were performed using the ‘caliper’ function of the Heidelberg instrument.

### Outcomes and statistical analyses

The primary outcome was postoperative BCVA. The best measurement at any visit during the follow-up period was collected for each patient. Comparison of the preoperative and postoperative values was performed using the Wilcoxon signed-rank test. Associations between preoperative measurements and postoperative BCVA were investigated using Spearman’s rank correlation coefficient. Further investigation was performed to identify factors associated with the achievement of a preferable prognosis after cataract surgery. The World Health Organization definition of normal vision is ≥0.5 decimal VA, which is equivalent to ≤0.3 logMAR. In accordance with this definition, eyes were divided into two groups based on postoperative logMAR: eyes with BCVA ≤ 0.3 logMAR and eyes with BCVA > 0.3 logMAR. Baseline clinical data were compared between the two groups. Categorical variables were compared using Fisher’s exact test, and continuous variables were compared using the Mann–Whitney U test. The receiver operating characteristic curve were used to determine correlations between the preoperative parameters and postoperative BCVA. The best cut-off value for each parameter was determined on the basis of Youden’s index. All statistical analyses were performed using IBM SPSS Statistics for Windows, Version 25.0 (IBM Corp., Armonk, NY, USA). A *P* value < 0.05 was considered significant.

## Results

### Patient profile and details of cataract surgery

This study included 38 eyes of 38 patients. The baseline demographic data are shown in Table 1. The mean (±standard deviation) age at surgery was 62.1 ± 11.8 years, and 22 patients were female. Among the patients, 30 had typical RP, 5 had pericentral RP, and 3 had sector RP. We conducted genetic testing in 13 patients and identified mutations in 3 patients. The results showed *EYS* mutations in one patient, *ROM1* mutation in one patient, and *CHM* mutations in one patient. Standard phacoemulsification was performed in all cases. Intraocular lens implantation was performed in 37 eyes. Among them, 27 eyes (73.0%) received NX-70 (Santen, Osaka, Japan). The remaining eyes received other intraocular lenses, including DCB00V, PCB00V, and ZCV (AMO, Santa Ana, CA, USA); XY-1 (HOYA Surgical Optics, Tokyo, Japan); SN6A and CNA0T0 (Alcon Laboratories, Fort Worth, TX, USA); and PN6A (Kowa, Tokyo, Japan). One eye was intentionally left aphakic because of high myopia. No intraoperative complications were observed. The mean follow-up period was 413 ± 224 days.

**Table 1:** Demographic data and preoperative measurements of patients.

	38 eyes of 38 patients
Age (years), mean ± SD	62.1 ± 11.8
Sex, *n* (female:male)	22:17
Preoperative logMAR BCVA	
Median (IQR)	0.52 (1.02)
Range	0.00–3.00
Horizontal EZ width (μm)	
Median (IQR)	783 (3739)
Range	0–9837
Vertical EZ width (μm)	
Median (IQR)	761 (4485)
Range	0–10 250
Average EZ width (μm)	
Median (IQR)	769 (4090)
Range	0–10 043
EZ disruption, *n* (%)	18 (47.4)
DRIL, *n* (%)	8 (21.2)

*SD* standard deviation, *logMAR* logarithm of the minimum angle of resolution, *BCVA* best-corrected visual acuity, *IQR* interquartile range, *EZ* ellipsoid zone, *DRIL* disorganisation of retinal inner layers.

### Preoperative evaluation

**Fig. 1:**
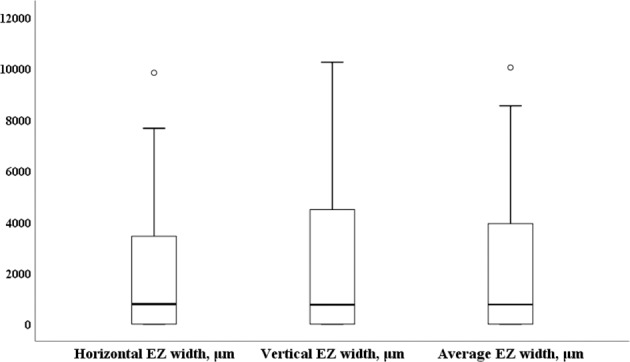
Distributions of the horizontal, vertical, and average ellipsoid zone (EZ) widths.

The median logMAR BCVA was 0.52 (range, 0.00–3.00; mean, 0.85 ± 0.84). The median horizontal EZ width was 783 μm (range, 0–9837); the median vertical EZ width was 761 μm (range, 0–10 250); and the median average EZ width was 769 μm (range, 0–10 043). The distributions of the horizontal, vertical, and average EZ width measurements are shown in Fig. 1. While 18 eyes (47.4%) had EZ disruption, 8 (21.2%) had DRIL.

### Outcomes

The median postoperative logMAR BCVA (the best value during the follow-up period) for all patients was 0.07 (range, −0.18–3.00; mean, 0.39 ± 0.72), and it significantly improved from the baseline (*P* < 0.001; Wilcoxon signed-rank test). While 25 eyes achieved a preferable visual prognosis (≤0.3 logMAR), 13 did not (>0.3 logMAR).

### Relationship between the preoperative evaluation and outcomes

**Fig. 2:**
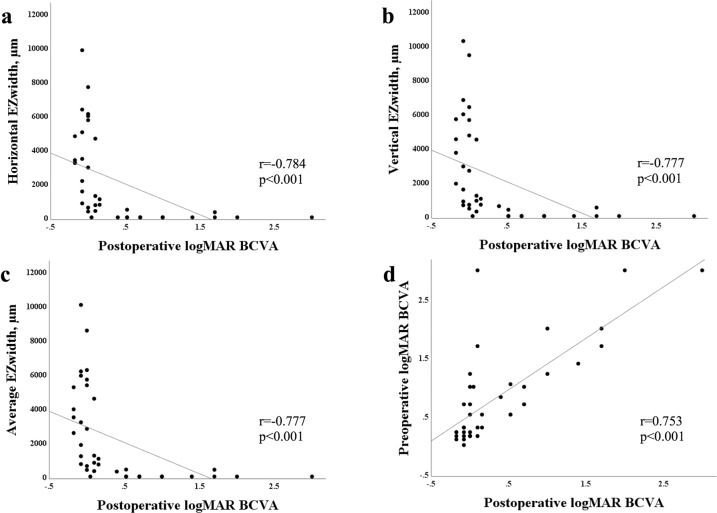
Scatter plots showing the relationships between preoperative measurements and postoperative logarithm of the minimum angle of resolution (logMAR) best-corrected visual acuity (BCVA). **a** Relationship between the horizontal ellipsoid zone (EZ) width and postoperative logMAR BCVA. **b** Relationship between the vertical EZ width and postoperative logMAR BCVA. **c** Relationship between the average EZ width and postoperative logMAR BCVA. **d** Relationship between preoperative and postoperative logMAR BCVA.

**Fig. 3:**
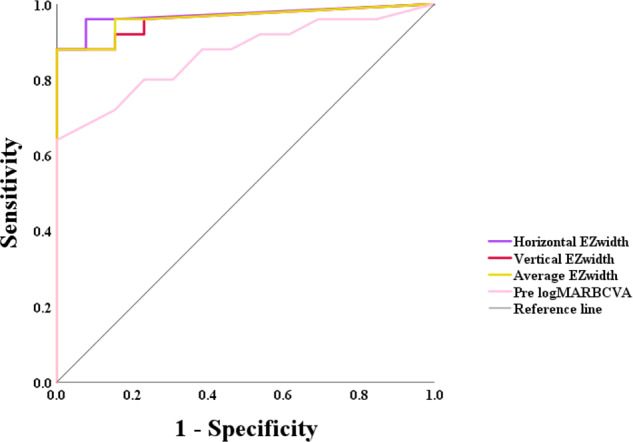
Receiver operating characteristic curves of the horizontal, vertical, and average ellipsoid zone (EZ) widths and preoperative logarithm of the minimum angle of resolution (logMAR) best-corrected visual acuity (BCVA).

Figure 2 shows a scatter plot of the preoperative measurements and postoperative logMAR BCVA. Postoperative logMAR BCVA significantly correlated with the horizontal (*r* = −0.784, *P* < 0.001), vertical (*r* = −0.777, *P* < 0.001), or average EZ widths (*r* = −0.777, *P* < 0.001), and preoperative logMAR BCVA (*r* = 0.753, *P* < 0.001). Shorter horizontal, vertical, and average EZ widths were associated with higher postoperative logMAR BCVA (lower vision). Higher preoperative logMAR BCVA was also associated with higher postoperative logMAR BCVA. Demographic characteristics and preoperative parameters of the patients with and without preferable prognoses are shown in Table 2. There was no significant difference in age and sex between both groups. The horizontal, vertical, and average EZ widths were significantly longer in eyes with BCVA ≤ 0.3 logMAR than in those with BCVA > 0.3 logMAR. Preoperative BCVA was significantly better in eyes with BCVA ≤ 0.3 logMAR than in those with BCVA > 0.3 logMAR. Both EZ disruption and DRIL were significantly more frequent in eyes with BCVA > 0.3 logMAR than in those with BCVA ≤ 0.3 logMAR. The area under the receiver operating characteristic curve (AUC) (Fig. 3) for the ability of the horizontal, vertical, and average EZ widths to discriminate eyes with postoperative BCVA ≤ 0.3 logMAR and eyes with BCVA > 0.3 logMAR was 0.971, 0.960, and 0.963, respectively. For preoperative logMAR BCVA, the AUC was 0.868. The best cut-off values for the horizontal, vertical, and average EZ widths were 513 μm (sensitivity, 0.880; specificity, 1.000), 608 μm (sensitivity, 0.880; specificity, 1.000), and 515 μm (sensitivity, 0.880; specificity, 1.000), respectively. For preoperative logMAR BCVA, the best cut-off value was 0.41 (sensitivity, 0.640; specificity, 1.000).

**Table 2:** Comparison of demographic data and preoperative measurements between eyes with BCVA ≤ 0.3 logMAR and eyes with BCVA > 0.3 logMAR.

	Postoperative BCVA		*P* value
	≤0.3 (25 eyes)	>0.3 (13 eyes)	
Age (years), mean ± SD	63.0 ± 12.2	62.1 ± 11.8	0.456
Sex, *n* (female:male)	25:15	8:12	0.084
Preoperative BCVA, median (IQR)	0.30 (0.54)	1.22 (1.24)	<0.001*
Horizontal EZ width (μm), median (IQR)	3198 (4582)	0 (0)	<0.001*
Vertical EZ width (μm), median (IQR)	2655 (4888)	0 (185)	<0.001*
Average EZ width (μm), median (IQR)	2795 (4738)	0 (145)	<0.001*
EZ disruption, *n* (%)	7 (28.0)	11 (84.6)	0.001*
DRIL, *n* (%)	2 (8.0)	6 (46.2)	0.011*

*BCVA* best-corrected visual acuity, *logMAR* logarithm of the minimum angle of resolution, *SD* standard deviation, *IQR* interquartile range, *EZ* ellipsoid zone, *DRIL* disorganisation of retinal inner layers.

*Significant at *P* < 0.05 (Mann–Whitney U test or Fisher’s exact test).

### Postoperative complications

The most common postoperative complication was posterior capsular opacification, which was detected in 10 eyes (26%) during the follow-up period, and neodymium: YAG laser capsulotomy was performed in all cases. Anterior capsule contraction was detected in three eyes (7.9%), and neodymium: YAG laser capsulotomy was performed in one case. One eye showed cystoid macular oedema on SD-OCT, and this eye was treated with topical non-steroidal anti-inflammatory drugs. No cases of intraocular lens dislocation or endophthalmitis were observed.

## Discussion

Our study reported the relationship between EZ width and visual prognosis after cataract surgery in patients with RP. We confirmed that the status of the preoperative EZ line was associated with postoperative BCVA. Although the presence of DRIL was also associated with postoperative BCVA, all eyes with DRIL in our study had concomitant EZ disruption. Preoperative EZ width significantly correlated with postoperative BCVA. Moreover, preoperative EZ width proved efficient in discriminating between eyes with postoperative BCVA ≤ 0.3 logMAR and those with BCVA > 0.3 logMAR. Measuring EZ width on preoperative SD-OCT images could be a useful tool for predicting the visual prognosis after cataract surgery in patients with RP.

Unstable predictability of visual prognosis is an important concern in cataract surgery for patients with RP. Some populations of patients may have a poor visual prognosis despite undergoing uncomplicated surgery. Patients with a preoperative VA of 20/400 or worse have been reported to have limited improvement in VA [3]. However, preoperative VA is not appropriate for predicting the visual prognosis because VA impairment is caused by both lenticular and retinal pathologies. Patients with a low VA may potentially show significant improvement, e.g., in cases of severe cataract with preserved central retinal function. Thus, the decision to perform cataract surgery should be based on the evaluation of the condition of the retina.

RP is characterised by the progressive loss of rod and cone photoreceptors, which begins in the peripheral retina and advances centripetally towards the fovea. Rod photoreceptors are primarily affected, and this is followed by the loss of secondary cone photoreceptors. A transition zone is known to exist between the healthy retina at the posterior pole and the unhealthy retina at the peripheral region. Observation of the transition zone has provided useful knowledge on the disease process. The earliest histopathological change is a shortening of the rod photoreceptor outer segment (PROS) [16]. OCT imaging has confirmed that the disease process begins with thinning of the PROS layer, which is followed by thinning of the outer nuclear layer, the disappearance of the EZ line, and disappearance of the external limiting membrane, eventually leading to the outer nuclear layer achieving an asymptotic thickness [17]. The EZ has been termed as the interface between the inner and outer segment junction of photoreceptors; however, recently, it has been considered to anatomically represent the ellipsoid component of the photoreceptors, which are packed with mitochondria [18]. In patients with RP, the disappearance of the EZ is considered to indicate an irreversible loss of the PROS layer in the transition zone. Thus, measuring EZ width enables the quantitative assessment of the remnant PROS. Cone photoreceptors are known to play a critical role in central vision. In typical cases of RP, cone photoreceptors tend to be preserved until extensive loss of rod photoreceptors has occurred. An early sign of cone photoreceptor damage is also a shortening of the PROS [19, 20]. Therefore, assessment of the PROS is considered important for estimating VA impairment.

The EZ offers several clinical advantages, including the ease and reliability in measuring its length. Strampe et al. reported excellent intra-observer repeatability and inter-observer reproducibility in measuring the length of the EZ line (EZ width) by manually marking the boundaries of the EZ band [21]. Moreover, a recent study applied artificial intelligence to evaluate EZ width in patients with RP [22]. Automated measurement of EZ width could have even more clinical applications.

In this study, the horizontal, vertical, and average EZ widths significantly correlated with BCVA after cataract surgery in patients with RP. Consistent with our results, Nakamura et al. reported that EZ width significantly correlated with postoperative BCVA [4]. Considering that VA mainly depends on cone photoreceptors in the central retina, longer than a certain length of EZ may not necessarily be required to preserve VA. Therefore, we additionally performed receiver operating characteristic analyses to discriminate between the eyes with and without a preferable prognosis after cataract surgery. The World Health Organization definition of normal vision (≥0.5 decimal VA) was used as the standard to judge the prognosis after surgery in this study. All of the horizontal, vertical, and average EZ widths had very high AUCs (above 0.95), which were superior to that of the preoperative BCVA (AUC = 0.868). In general, an AUC above 0.90 indicates high accuracy [23]. We proposed EZ width as a useful tool to predict the prognosis of cataract surgery in patients with RP.

The best cut-off values of EZ width for determining whether the patients achieved a preferable prognosis were identified as 513, 608, and 515 μm for the horizontal, vertical, and average values, respectively. Therefore, our results suggest that cataracts in patients with RP, EZ widths of which are preserved at approximately 600 μm, are good candidates for surgery. In this study, we focused on VA measurements to evaluate the visual prognosis. A decimal VA of 0.5 is generally considered the threshold for a motorbike driving license or good reading [24]. We should, however, note that patients with typical RP have poor contrast sensitivity because of their reduced visual fields, as well as poor dark/light adaptation even with a good VA. Thus, the World Health Organization definition of normal vision (≥0.5 decimal VA) may not be entirely applicable to patients with RP. Moreover, posterior subcapsular cataracts, which are the most common morphologic category in patients with cataract [25], could cause increased glare sensitivity regardless of VA [26]. Jackson et al. reported that most patients with RP experienced subjective improvements in visual symptoms even without significant improvement in VA after cataract surgery [2]. Notably, an EZ width of less than approximately 600 μm does not necessarily make cataract surgery inadvisable.

Among the limitations of our study are its retrospective design and limited sample size. Furthermore, SD-OCT measurements were performed manually. Future studies involving a larger sample size with automated computerised measurements are needed to verify the current results. In our patients, the most common postoperative complication was posterior capsular opacification, which was treated using neodymium: YAG laser capsulotomy in all cases. Nevertheless, patients with RP should be provided an adequate explanation about posterior capsular opacification and neodymium: YAG laser capsulotomy before the cataract surgery.

In conclusion, EZ width measured on SD-OCT images was associated with the visual prognosis after cataract surgery in patients with RP. We demonstrated that preoperative EZ width was efficient in discriminating between the eyes with and without a preferable prognosis. Restoration of normal vision in terms of VA can be expected in patients who have an EZ width of approximately 600 μm. Further investigations, including not only the assessment of VA but also the evaluations of other visual functions, are required for a comprehensive assessment of visual prognosis after cataract surgery in patients with RP.

## Summary

### What was known before

- Some populations of patients with retinitis pigmentosa who underwent cataract surgery could not achieve improvement in visual acuity.
- The integrity of the ellipsoid zone line has been reported as important for the prediction of visual prognosis after cataract surgery in patients with retinitis pigmentosa.

### What this study adds

- Preoperative ellipsoid zone width was found to be efficient in discriminating between the eyes with and without a preferable visual prognosis.
- Restoration of normal vision in terms of visual acuity can be expected in patients with an ellipsoid zone width of approximately 600 μm.
